# Transcatheter arterial chemoembolization of hepatocellular carcinoma in a patient with celiac trunk occlusion: a therapeutic challenge

**DOI:** 10.1590/1677-5449.180090

**Published:** 2019-05-22

**Authors:** Altino Ono Moraes, Ecio Alves do Nascimento, Tiago Francisco Meleiro Zubiolo, Marcos Fábio Maximiano de Paula, Augusto Felipe Bruchez Brito, Bruno Filipe Viotto Petta, Gustavo Martini Perini, Dariane Rosa Martins

**Affiliations:** 1 Instituto de Moléstias Vasculares – IMV, Maringá, PR, Brasil; 2 Hospital Universitário Regional de Maringá, Maringá, PR, Brasil.; 3 Hospital Santa Rita de Maringá, Maringá, PR, Brasil.; 4 Centro Universitário Ingá – UNINGÁ, Departamento de Medicina, Maringá, PR, Brasil.

**Keywords:** chemoembolization, hepatocellular carcinoma, palliative treatment

## Abstract

Transcatheter arterial chemoembolization is a technique for provoking ischemia and cytotoxic activity by selectively injecting microspheres containing chemotherapy drugs into vessels supplying a tumor. An 87-year-old female patient on palliative treatment for hepatocellular carcinoma and with indications for chemoembolization underwent preparatory angiography, which revealed celiac trunk occlusion. The treatment option chosen was selective catheterization of the hepatic artery proper to release the chemotherapy agent via an alternative route through the superior mesenteric artery with communication using the pancreaticoduodenal arcade. Studies have reported evidence showing increased survival after chemoembolization and also reduced tumor growth rate. However, difficulties with accessing and catheterizing the artery feeding the tumor via the alternative access make the procedure a challenge, because of the tortuosity of the pancreaticoduodenal arcade.

## INTRODUCTION

Hepatocellular carcinoma (HCC) is the most common primary liver tumor, with elevated morbidity and mortality rates all over the world.^1^ Although surgical resection is a curative treatment, few cases are candidates for hepatectomy, because of advanced multifocal disease, extrahepatic tumoral invasion, deterioration of liver function, portal hypertension, and poor functional status.^1^^,^ 2 Liver transplantation is another effective option for curative treatment, but it is limited to the following cases: one tumor less than 5 cm in diameter or three tumors less than 3 cm in diameter (Milan Criteria), in addition to difficulties with finding donors.^1^

Transcatheter arterial chemoembolization (TACE) is currently used as a palliative treatment for patients with tumors that cannot be resected^3^^,^ 4 and also in cases of resectable tumors, to reduce tumor volume and dispersal of neoplastic cells during manipulation in surgical procedure.^2^ The mechanism through which TACE achieves its objective is induction of ischemia in the tumor (generally by injection of gelfoam or microspheres) through the cytotoxic action of a chemotherapy agent selectively administered with the aim of provoking long-lasting intratumoral retention. This therapeutic method has a high positive response rate, retarding tumor progression and increasing survival. Once injected, the chemotherapy drug should ideally be retained in the tumor and released gradually to avoid systemic toxicity.^5^ Currently, the drugs most often used are doxorubicin, combined or not with mitomycin or cisplatin, and irinotecan. There are two methods of chemoembolization in use: either conventional, using a chemotherapy mixture (with or without a liposoluble vehicle) combined with permanent or temporary emboligenic materials; or with granules loaded with a chemotherapy drug, using emboligenic microspheres.^6^

### Part I – Clinical situation

The patient was an 87-year-old female with hypertension and long-term diabetes. She had started seeing a hepatologist in 2009 because of a liver nodule found incidentally during a computed tomography examination of the abdomen, located in segment VI, with a diameter of approximately 2.6 cm, and no specific characteristics visible. During the initial investigation, renal and hepatic functions were normal, viral hepatitis serologies were negative, and alpha-fetoprotein was within normal limits. After 2 years, a control tomography showed a discrete enlargement in the nodule located in segment VI (2.8 cm) and a second nodule that had appeared in segment VII (0.9 cm).

During 2011, a repeat tomography showed that the nodule had enlarged further and, for the first time, a contrast uptake pattern was seen during the arterial phase, with washout during the other phases, compatible with a diagnosis of HCC, differentiating it from any type of benign lesion. In view of the patient’s comorbidities, the lesion was not biopsied and the patient was referred to our service for chemoembolization with doxorubicin, as a palliative measure, to prolong survival and quality of life.

Arteriography conducted preparatory to chemoembolization revealed obstruction of the celiac trunk, probably caused by atherosclerotic disease, making it a challenge to reach the tumor.

A total of four embolizations with doxorubicin were performed, in 2011, 2012, 2014, and, the most recent, in May of 2018. Control examinations showed an average regression of 30-40% in the diameter of the primary tumor. However, as the disease advanced, smaller-diameter satellite lesions began to appear, creating additional obstacles to palliative treatment. Nevertheless, after 9 years’ follow-up the patient still has preserved liver function and there is no evidence of thrombosis of the adjacent portal vein.

### Part II – What was done

**Figure 1:**
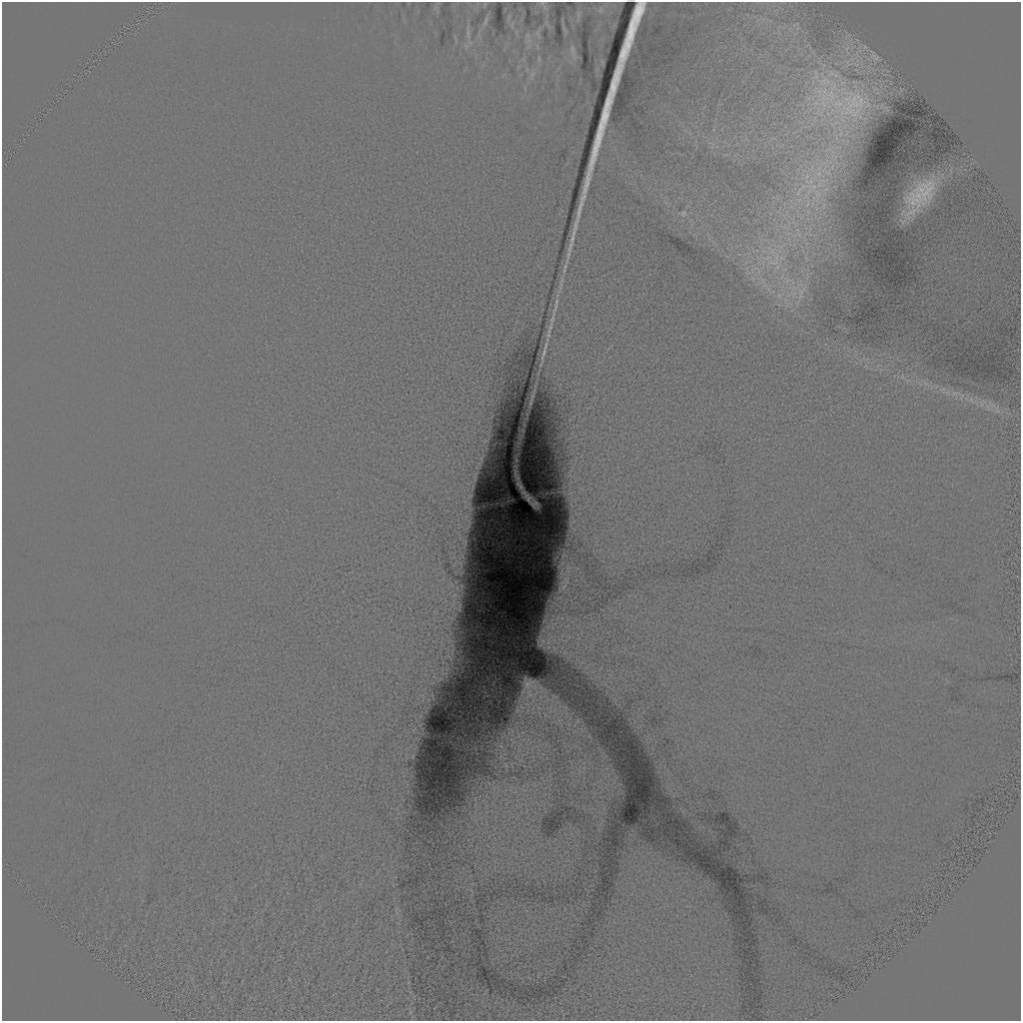
Aortography showing subocclusion of the celiac trunk.

**Figure 2:**
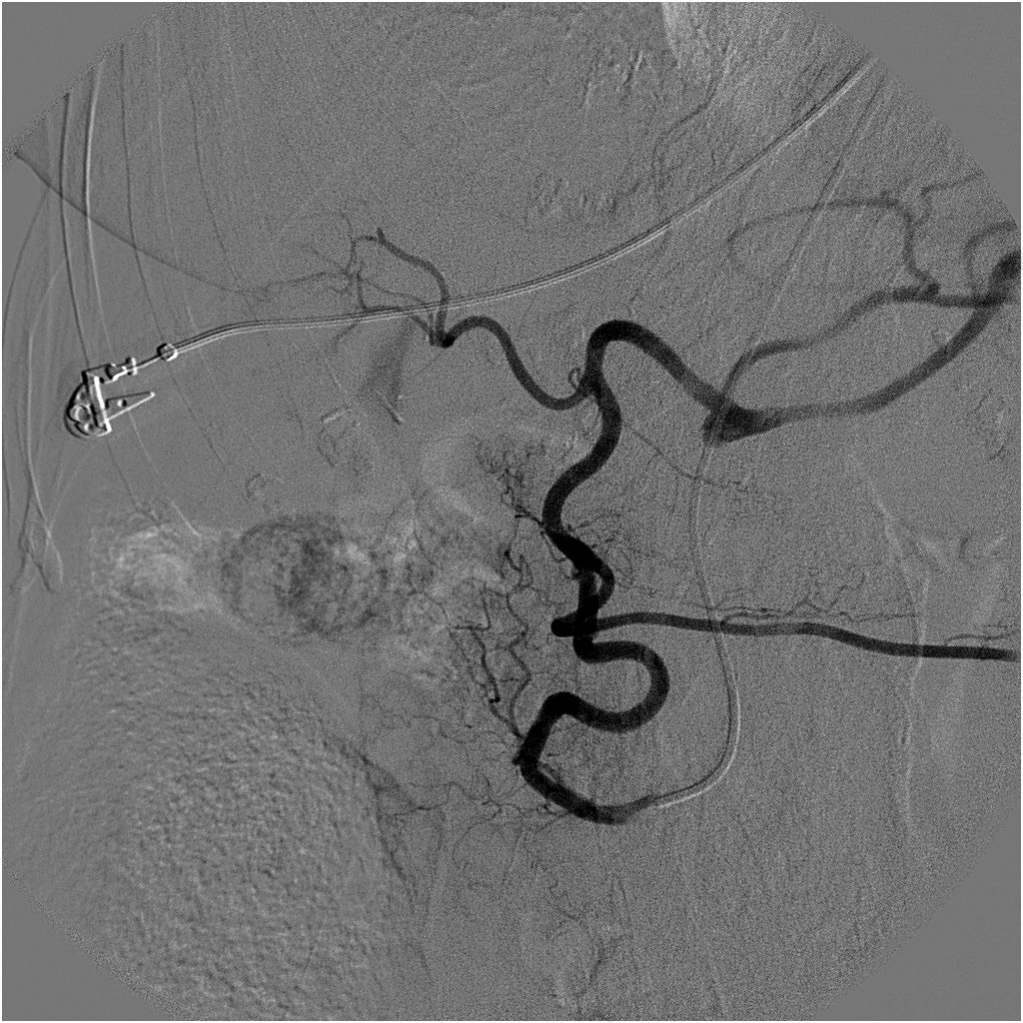
Catheterization of the pancreaticoduodenal arcade via the superior mesenteric artery and access to the hepatic artery proper.

The procedures were all similar, but we will only describe the most recent, performed in 2018. With the patient under general anesthesia and aseptic technique, ultrasound-guided puncture of the right radial artery was performed, followed by placement of a 6F introducer specific for the radial artery, insertion of a 0.035 hydrophilic guidewire and multipurpose catheter, and catheterization of the abdominal aorta. Contrast imaging confirmed obstruction of the celiac trunk and patency of the superior mesenteric artery, an alternative route to access the liver (Figure 1). After catheterization of the superior mesenteric artery, the 0.035 guidewire was substituted for a 0.014 guidewire with microcatheter (Figure 2).

**Figure 3:**
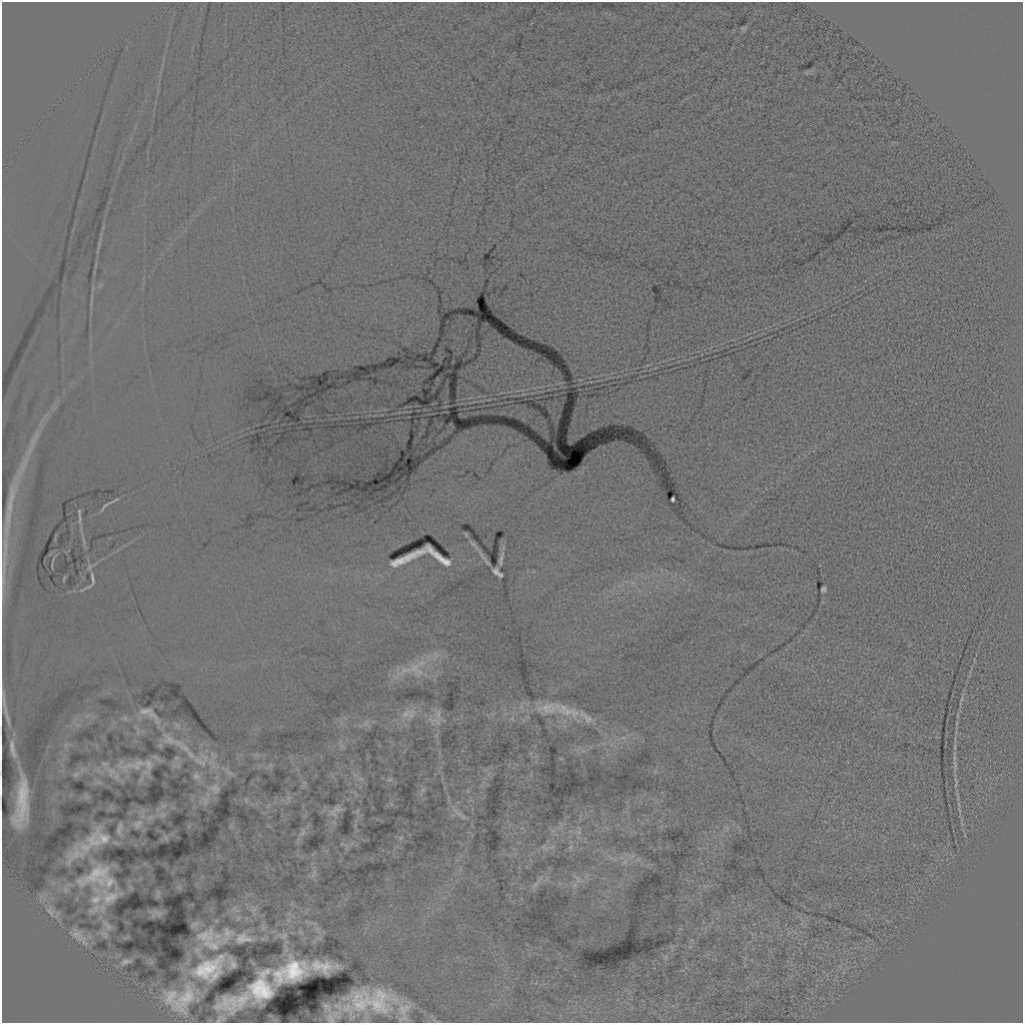
Superselective catheterization of intrahepatic arteries feeding the tumor.

**Figure 4:**
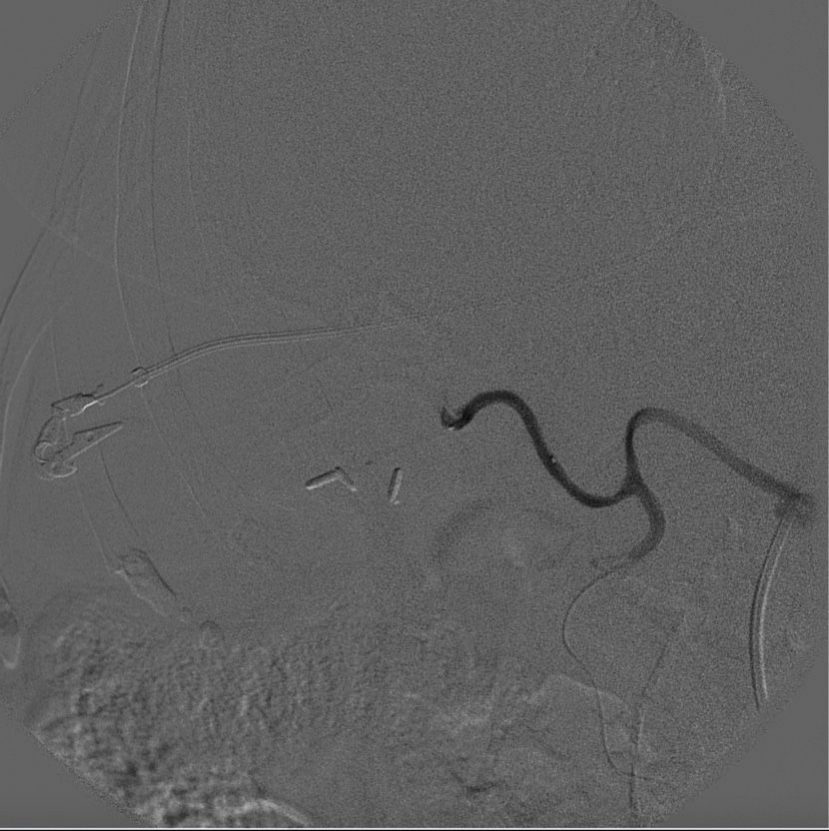
Control arteriography showing satisfactory embolization of arteries that feed the tumor.

The alternative route was, in the following order, via the inferior and superior pancreaticoduodenal arteries, the gastroduodenal artery, the hepatic artery proper, and the right hepatic artery. After superselective catheterization of the topography of segment VI (Figure 3), which was the site of the largest nodule and the greatest number of satellite nodules, 150-200 µm Hepaspheres™ (Merit Medical Systems, United States) loaded with doxorubicin were delivered. A control arteriography demonstrated satisfactory embolization of the target vessels (Figure 4).

## DISCUSSION

Since the start of the century, diabetes mellitus has been considered a risk factor for emergence of several types of cancer. El-Serag et al. conducted a meta-analysis with 13 cohort studies and 13 case-control studies that linked the disease with a 2.5 times increased risk of appearance of hepatocarcinoma. Other studies confirmed these findings, attributing from 2 to 3 times increased risk and a considerable proportion of these studies only included patients with negative serology for viral hepatitis.^7^

The complex process of carcinogenesis involves several stages, that can be summarized as follows: endogenous hyperinsulinemia (insulin resistance), exogenous hyperinsulinemia (treatment with insulin or secretagogues), hyperglycemia, increased proinflammatory state and oxidative stress make damage to the genetic code more likely, increased angiogenesis and cell proliferation and reduced apoptosis rate.^8^ Therefore, since diabetes is a risk factor for hepatic neoplasms and our patient had negative serology for viral hepatitis, the fact that she had had diabetes for a long time supports the likelihood of a relationship between the two diseases.

In order to perform selective chemoembolization of HCC, it is necessary to catheterize the hepatic arteries via the celiac trunk, in order to determine which vessels lead to the lesion. This involves conducting an anatomovascular study of the tumor using the conventional arteriography technique with contrast to observe the origin of the HCC and the tortuosity of adjacent vessels and arteries, in addition to detecting anatomic variations, stenoses, transmural atherosclerotic disease, and possible occlusions of the celiac trunk and superior mesenteric artery. A microcatheter is inserted through the catheter to administer the injection of granules containing chemotherapy agents only into the vessels feeding the tumor, thereby avoiding necrosis of healthy tissue. At the end of TACE, control angiography is performed to confirm total devascularization of the target site.^6^

**Figure 5:**
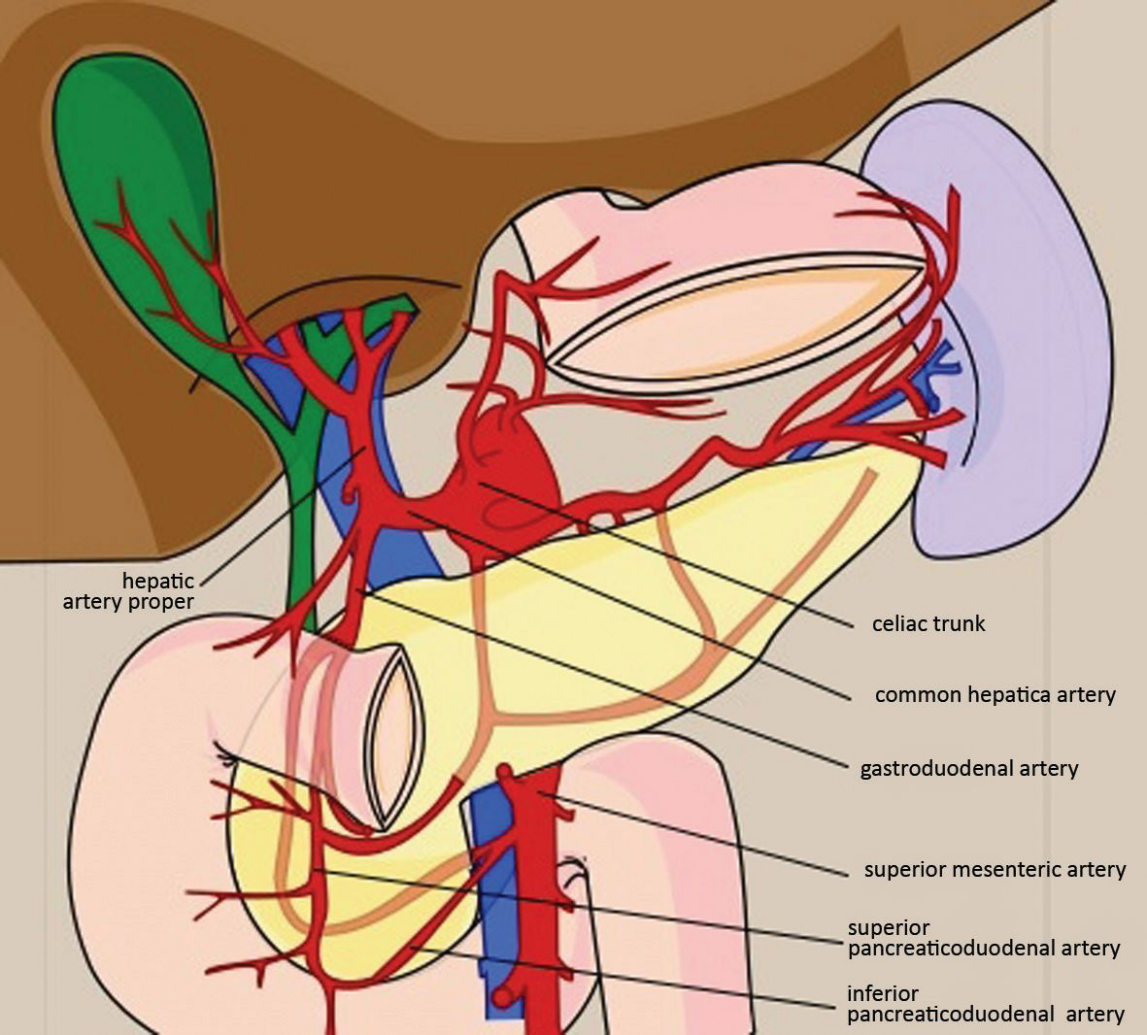
Schematic diagram illustrating arteries related to the tumor and the alternative route employed during the procedure.

In the case described here, the traditional access via the celiac trunk was not possible. Compression by the median arcuate ligament of the diaphragm tends to be identified as the cause of the majority of celiac axis occlusions. However, using imaging methods we identified that there was an intimate relationship with an atherosclerotic process. According to clinical trials, catheterization of arteries occluded by extrinsic compression is easier than catheterization of arteries occluded intrinsically, such as by atherosclerosis.^2^ When the usual access cannot be obtained, an alternative approach is possible via the superior mesenteric artery to the hepatic artery proper, by dilation of the pancreaticoduodenal arcade and the gastroduodenal artery^9^ (Figure 5).

Recent studies have found significant results in terms of increased survival of patients with HCC after chemoembolization. A systematic review conducted in 2016 by Lencioni et al., including 10,108 patients in 101 studies, evaluated that this treatment resulted in a tumor response rate of 52.5% and mean survival of 19.4 months, with 70.3% at 1 year, 40.4% at 3 years, and 34.4% at 5 years.^4^

Observational studies conducted on a global scale concluded that, to date, approximately half of patients with HCC have been given TACE at some point of their treatment. Meta-analysis studies have identified an evident increase in survival among patients treated with TACE using lipiodol, cisplatin, or doxorubicin, when compared with conservative treatment.^4^

In a safety review, a total of 214 deaths were recorded in a study with 34,137 patients who underwent 50,953 transcatheter arterial chemoembolizations, resulting in an overall mortality rate of 0.6%, while the most common adverse events were liver failure (n = 59), followed by infectious complications (n = 20), gastrointestinal bleeding (n = 17), and rupture of intraperitoneal tumors (n = 8).^4^

In a study conducted by Kwon et al., it did not prove possible to catheterize the hepatic artery proper via the alternative access in 8% of the patients. Problems mentioned were difficulties with manipulation of the catheter because of the tortuosity of the aorta, the small caliber and tortuosity of the pancreaticoduodenal arcade and the acute angle between the hepatic artery proper and the gastroduodenal artery. These cases were managed with percutaneous ethanol injection, systemic chemotherapy, and surgical operations. In one of these cases, vascular dissection of the celiac trunk was described; however, since this was not a major complication, the patient underwent TACE successfully and at the next session the rupture had resolved.^2^

In another case report, published by Geiger et al.^10^, a patient with stenosis of the celiac trunk had a 6 x 15 mm stent deployed successfully via the superior mesenteric artery, which furnished the vascular surgeon with a new access route to the hepatic arteries and the possibility of less invasive, although difficult to execute, treatment.^10^

We conclude that this was a challenging case in which obstruction of the celiac trunk – the traditional access route to the hepatic arteries, led us to employ an alternative route with greater tortuosity and technical difficulty, but which successfully achieved the objective. Another relevant detail of this case is the survival of the patient who, despite being in her ninth decade of life and undergoing palliative treatment, has satisfactory quality of life.
