# Supplementary material for: Transcatheter arterial chemoembolization of hepatocellular carcinoma in a patient with celiac trunk occlusion: a therapeutic challenge
Source: J Vasc Bras. 2019 May 22;18:e20180090. doi: 10.1590/1677-5449.180090 (PMC6634952; doi:10.1590/1677-5449.180090)
Supplement: Translation

# Translation: Quimioembolização arterial transcateter de carcinoma hepatocelular em paciente com oclusão de tronco celíaco: um desafio terapêutico

**Authors:** Altino Ono Moraes, Ecio Alves do Nascimento, Tiago Francisco Meleiro Zubiolo, Marcos Fábio Maximiano de Paula, Augusto Felipe Bruchez Brito, Bruno Filipe Viotto Petta, Gustavo Martini Perini, Dariane Rosa Martins

**Affiliations:** 1 Instituto de Moléstias Vasculares – IMV, Maringá, PR, Brasil.; 2 Hospital Universitário Regional de Maringá, Maringá, PR, Brasil.; 3 Hospital Santa Rita de Maringá, Maringá, PR, Brasil.; 4 Centro Universitário Ingá – UNINGÁ, Departamento de Medicina, Maringá, PR, Brasil.

**Keywords:** quimioembolização, carcinoma hepatocelular, tratamento paliativo

## Abstract

A quimioembolização arterial transcateter consiste em promover isquemia e ação citotóxica por meio da injeção de microesferas contendo quimioterápicos aplicada seletivamente nos vasos que nutrem o tumor. Paciente do sexo feminino, com 87 anos, em tratamento paliativo para carcinoma hepatocelular com indicação de quimioembolização, foi submetida a angiografia pré-procedimento, a qual identificou oclusão do tronco celíaco. A opção foi a cateterismo seletiva da artéria hepática própria por uma via alternativa para lançamento do agente quimioterápico, através da artéria mesentérica superior com comunicação utilizando a arcada pancreatoduodenal. Evidências de aumento da sobrevida demonstrados em estudos após a quimioembolização foram relatados, bem como redução da taxa de crescimento tumoral; entretanto, dificuldades em acessar e cateterizar a artéria nutrícia tumoral através do acesso alternativo tornam o procedimento um desafio, devido à tortuosidade da arcada pancreatoduodenal.

## INTRODUÇÃO

Carcinoma hepatocelular (CHC) é o principal tumor primário de fígado e tem elevada morbimortalidade em todo o mundo^1^. Embora a ressecção cirúrgica represente um tratamento curativo, poucos casos são candidatos a hepatectomia, devido à existência de doença multifocal avançada, invasão tumoral extra-hepática, deterioração da função hepática, hipertensão portal e status funcional ruim^1^^,^ 2. O transplante hepático é uma opção efetiva para o tratamento curativo, porém é limitado aos seguintes casos: um tumor com menos de 5 cm de diâmetro ou três tumores com menos de 3 cm de diâmetro (Critérios de Milão), além de haver dificuldade de obtenção de doador^1^.

A quimioembolização arterial transcateter (*transcatheter arterial chemoembolization*, TACE) é atualmente usada como tratamento paliativo em pacientes com tumores irressecáveis^3^^,^ 4 e também nos casos de tumores ressecáveis, para reduzir seu volume e prevenir a dispersão de células neoplásicas durante manipulação em procedimento cirúrgico^2^. A TACE atinge seu objetivo por induzir isquemia tumoral (geralmente pela injeção de gelfoam ou microesferas) devido à ação citotóxica do quimioterápico administrado seletivamente visando retenção intratumoral duradoura. Esse método terapêutico apresenta alta taxa de resposta positiva, promovendo retardo na progressão tumoral e aumento da sobrevida. Idealmente, o quimioterápico injetado deve ser retido pelo tumor e liberado gradualmente, para evitar toxicidade sistêmica^5^. As drogas mais utilizadas atualmente são doxorrubicina, associada ou não com mitomicina ou cisplatina, e irinotecano. Duas modalidades de quimioembolização podem ser encontradas: convencional, utilizando uma mistura de quimioterápico (com ou sem carreador lipossolúvel) associada com materiais emboligênicos permanentes ou temporários; ou com grânulos preenchidos com quimioterápico, utilizando microesferas emboligênicas^6^.

### Parte I – Situação clínica

Paciente do sexo feminino, de 87 anos, hipertensa e diabética de longa data, iniciou acompanhamento com hepatologista no ano de 2009 devido a um achado incidental de nódulo hepático em exame de tomografia computadorizada de abdome, com aproximadamente 2,6 cm de diâmetro, no segmento VI, não sendo possível visualizar características específicas. Durante investigação inicial, funções hepática e renal normais, sorologias para hepatites virais negativas e alfafetoproteína dentro da normalidade. Após 2 anos, tomografia de controle revelou discreto aumento no nódulo localizado no segmento VI (2,8 cm) e aparecimento de um segundo nódulo no segmento VII (0,9 cm).

No ano de 2011, nova tomografia evidenciou aumento do nódulo e, pela primeira vez, foi identificado um padrão de captação de contraste na fase arterial e *washout* (rápida perda de realce de contraste) nas outras fases, o que corrobora com o diagnóstico de CHC, diferenciando-o de qualquer outro tipo de lesão benigna. Devido a comorbidades, não foi realizada biópsia da lesão e a paciente foi encaminhada para nosso serviço com o objetivo de realizar quimioembolização com doxorrubicina como medida paliativa, objetivando prolongar a sobrevida e a qualidade de vida.

Realizada previamente à quimioembolização, arteriografia identificou obstrução do tronco celíaco por provável doença aterosclerótica, tornando-se um desafio atingir o tumor.

Ao longo do tempo, foram realizadas quatro embolizações com doxorrubicina, nos anos 2011, 2012, 2014 e, a última, em maio de 2018. Exames de controle mostraram regressão de, em média, 30-40% do diâmetro do tumor primário; porém, com o avanço da doença, ocorreu o aparecimento de lesões-satélite de menor diâmetro, sendo um obstáculo maior ao tratamento paliativo. Contudo, após 9 anos de acompanhamento, a paciente mantém sua função hepática preservada, sem evidência de trombose de veia porta adjacente.

### Parte II – O que foi feito

Os procedimentos realizados foram semelhantes; portanto, descreveremos apenas o último, realizado no ano de 2018. Com a paciente em anestesia geral com técnica asséptica, foram realizadas punção ecoguiada de artéria radial direita, colocação de introdutor 6F específico para artéria radial, passagem de fio-guia hidrofílico 0,035 e cateter multipurpose, e cateterismo de aorta abdominal. Exame contrastado confirmou obstrução do tronco celíaco e perviedade da artéria mesentérica superior, uma via alternativa de acesso ao fígado (Figura 1). Após cateterismo da artéria mesentérica superior, o fio-guia 0,035 foi substituído pelo fio-guia 0,014 associado ao microcateter (Figura 2).

**Figura 1:** Aortografia evidenciando suboclusão do tronco celíaco.

**Figura 2:** Cateterismo da arcada pancreaticoduodenal via artéria mesentérica superior e acesso a artéria hepática própria.

O caminho alternativo foi, nesta ordem, via artérias pancreaticoduodenais inferior e superior, artéria gastroduodenal, artéria hepática própria e artéria hepática direita. Após cateterismo superseletiva na topografia do segmento VI (Figura 3), local onde estava o maior nódulo e a maior quantidade de nódulos-satélite, foi entregue a droga doxorrubicina associada a Hepashperas™ 150-200 µm (Merit Medical Systems, EUA). Arteriografia de controle demonstrou embolização satisfatória dos vasos-alvo (Figura 4).

**Figura 3:** Cateterismo superseletiva de artérias intra-hepáticas que nutriam o tumor.

**Figura 4:** Arteriografia de controle mostrando embolização satisfatória das artérias que nutriam o tumor.

## DISCUSSÃO

Desde o início do século, o diabetes melito é considerado um fator de risco para o aparecimento de vários tipos de câncer. El-Serag et al. conduziram uma metanálise com 13 estudos de coorte e 13 estudos tipo caso-controle que relacionaram a doença ao aparecimento de hepatocarcinoma, com risco aumentado em 2,5 vezes. Outros estudos corroboram com os achados, atribuindo um risco aumentado variando entre 2 a 3 vezes, sendo que boa parte dos estudos incluiu somente pacientes com sorologia negativa para hepatites virais^7^.

O complexo processo de carcinogênese envolve vários estágios, de forma que podemos resumir da seguinte maneira: hiperinsulinemia endógena (resistência insulínica), hiperinsulinemia exógena (tratamento com insulina ou secretagogos), hiperglicemia, aumento do estado pró-inflamatório e do estresse oxidativo favorecem dano ao código genético, aumento na angiogênese e na proliferação celular e redução na taxa de apoptose^8^. Portanto, visto que o diabetes é um fator de risco para neoplasia hepática e nossa paciente tinha sorologias negativas para hepatites virais, o fato de ser diabética de longa data corrobora com a relação de ambas as doenças.

Para a realização da quimioembolização seletiva do CHC, é necessária a cateterismo das artérias hepáticas através do tronco celíaco, visando conhecer os vasos que vão até a lesão. Para isso, se faz necessário um estudo anatomovascular do tumor utilizando a técnica de arteriografia convencional com contraste para estudo da origem do CHC e da tortuosidade dos vasos e artérias adjacentes, além de detecção de variações anatômicas, estenoses, doença aterosclerótica transmural e possíveis oclusões do tronco celíaco e da artéria mesentérica superior. Um microcateter é introduzido por meio do cateter para aplicar a injeção dos grânulos contendo quimioterápicos apenas nos vasos que nutrem o tumor, evitando assim a necrose de tecido sadio. Marcando o fim da TACE, é realizada uma angiografia de controle para confirmar a total desvascularização do local-alvo^6^.

No caso em questão, não foi possível o acesso tradicional via tronco celíaco. A compressão do ligamento arqueado mediano do diafragma costuma ser apontada como a causa de compressão da maioria das oclusões do eixo celíaco; porém, identificamos, por métodos de imagem, íntima relação com processo aterosclerótico. Segundo ensaio clínico, a cateterismo de artérias ocluídas por compressão extrínseca é mais fácil do que a de artérias ocluídas intrinsecamente, como pela aterosclerose^2^. Quando o acesso usual não for possível, o caminho alternativo é obtido através da artéria mesentérica superior para a artéria hepática própria, por meio de dilatação da arcada pancreaticoduodenal e da artéria gastroduodenal^9^ (Figura 5).

**Figura 5:** Desenho esquemático das artérias relacionadas ao tumor e do caminho alternativo utilizado durante o procedimento.

Estudos recentes encontraram resultados significativos em relação ao aumento da sobrevida nos pacientes com CHC após quimioembolização. Uma revisão sistemática realizada em 2016 por Lencioni et al. incluindo 10.108 pacientes em 101 estudos avaliou que o tratamento resultou em uma taxa de resposta tumoral de 52,5% e sobrevida média de 19,4 meses, sendo 70,3% no primeiro ano, 40,4% no terceiro ano e 34,4% no quinto ano^4^.

Estudos observacionais em escala global concluíram que, até agora, aproximadamente metade dos pacientes com CHC, em algum momento de seu tratamento, recebem TACE. Estudos de metanálise identificaram um evidente aumento na sobrevida em pacientes submetidos a TACE com lipidol, cisplatina ou doxorrubicina, quando comparados com tratamento conservador^4^.

Em uma revisão de segurança, foram notados 214 óbitos num estudo envolvendo 34.137 pacientes que foram submetidos a 50.953 quimioembolizações arteriais transcateter, resultando em uma taxa de mortalidade geral de 0,6%, sendo que a maior parte dos eventos adversos foi falência hepática (n = 59), seguido por complicações infecciosas (n = 20), sangramento gastrointestinal (n = 17) e ruptura de tumor intraperitoneal (n = 8)^4^.

Em um estudo realizado por Kwon et al., não foi possível cateterizar a artéria hepática própria através do acesso alternativo em 8% dos pacientes. A adversidade mencionada foi a difícil manipulação do cateter devido à tortuosidade da aorta, ao pequeno calibre e tortuosidade da arcada pancreatoduodenal e ainda ao ângulo agudo entre a artéria hepática própria e a artéria gastroduodenal. As condutas seguidas nesses casos foram de injeção de etanol percutânea, quimioterapia sistêmica e operação cirúrgica. Em um dos casos, foi relatada a dissecção vascular do tronco celíaco; entretanto, como tratava-se de uma complicação menor, o paciente foi submetido ao TACE com sucesso e em sua próxima sessão a ruptura estava resolvida^2^.

Em outro relato de caso realizado por Geiger et al.^10^, uma paciente com estenose de tronco celíaco teve um stent de 6 x 15 mm implantado com sucesso via artéria mesentérica superior, o que acrescenta ao cirurgião vascular uma nova rota de acesso às artérias hepáticas e a possibilidade de ampliação da terapêutica menos invasiva, mas de difícil realização^10^.

Diante do exposto, concluímos que se tratou de um caso desafiador, no qual a obstrução do tronco celíaco, via tradicional de acesso às artérias hepáticas, levou-nos a utilizar uma via alternativa com maior tortuosidade e dificuldade técnica, porém com êxito no objetivo proposto. Outro detalhe relevante do caso foi a sobrevida da paciente que, mesmo em sua nona década de idade e passando por um tratamento paliativo, tem qualidade de vida satisfatória.
